# Supplementary material for: ERAP1 promotes Hedgehog-dependent tumorigenesis by controlling USP47-mediated degradation of βTrCP
Source: Nat Commun. 2019 Jul 24;10:3304. doi: 10.1038/s41467-019-11093-0 (PMC6656771; doi:10.1038/s41467-019-11093-0)
Supplement: Supplementary file 1 — Supplementary Information [file 41467_2019_11093_MOESM1_ESM.pdf]

ERAP1 promotes Hedgehog-dependent tumorigenesis by controlling USP47-mediated degradation of βTrCP

Bufalieri et al.

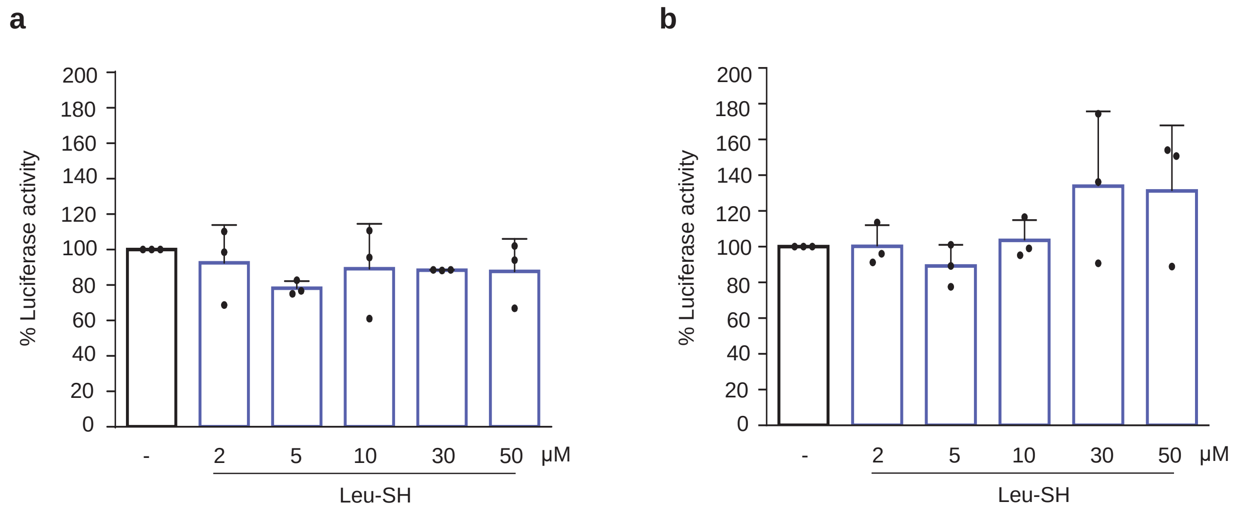

Supplementary Figure 1. **(a,b)** ERAP1 inhibition does not affect Jun or Wnt pathways. Luciferase activity of MEF cells transfected with MMP1 luciferase reporter and C-Jun (a) or Top Flash luciferase reporter and β-Catenin (b) and treated with increasing amounts of Leu-SH or DTT as control. Data are normalized against *Renilla* activity and indicated as percentage relative to control. Statistical significance was determined with two-tailed Student’s *t*-test.

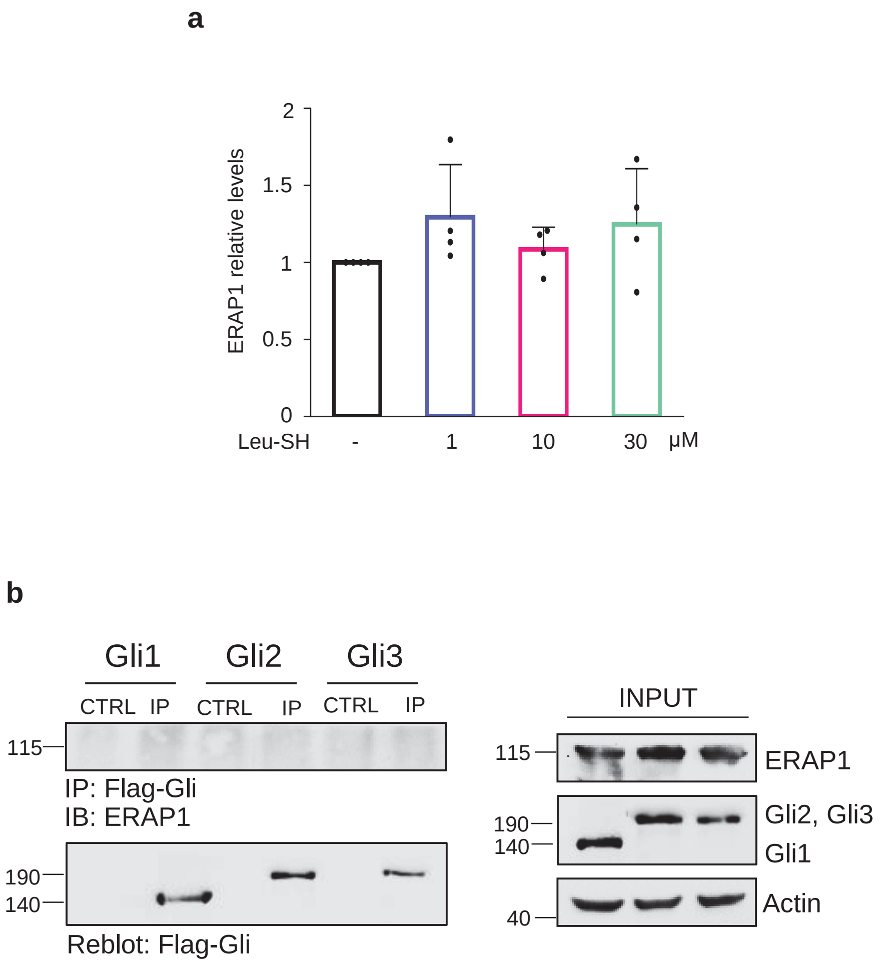

Supplementary Figure 2. **(a)** Densitometric analysis of ERAP1 protein levels after treatment with Leu-SH. Values represent the mean of more than three independent experiments. Statistical significance was determined with two-sided Student's *t*-test. **(b)** MEFs were transfected with ERAP1 and Flag-Gli1 or Flag-Gli2 or Flag-Gli3 as indicated. Cell lysates were immunoprecipitated with an anti-Flag antibody and immunoblotted with anti-ERAP1 antibody.

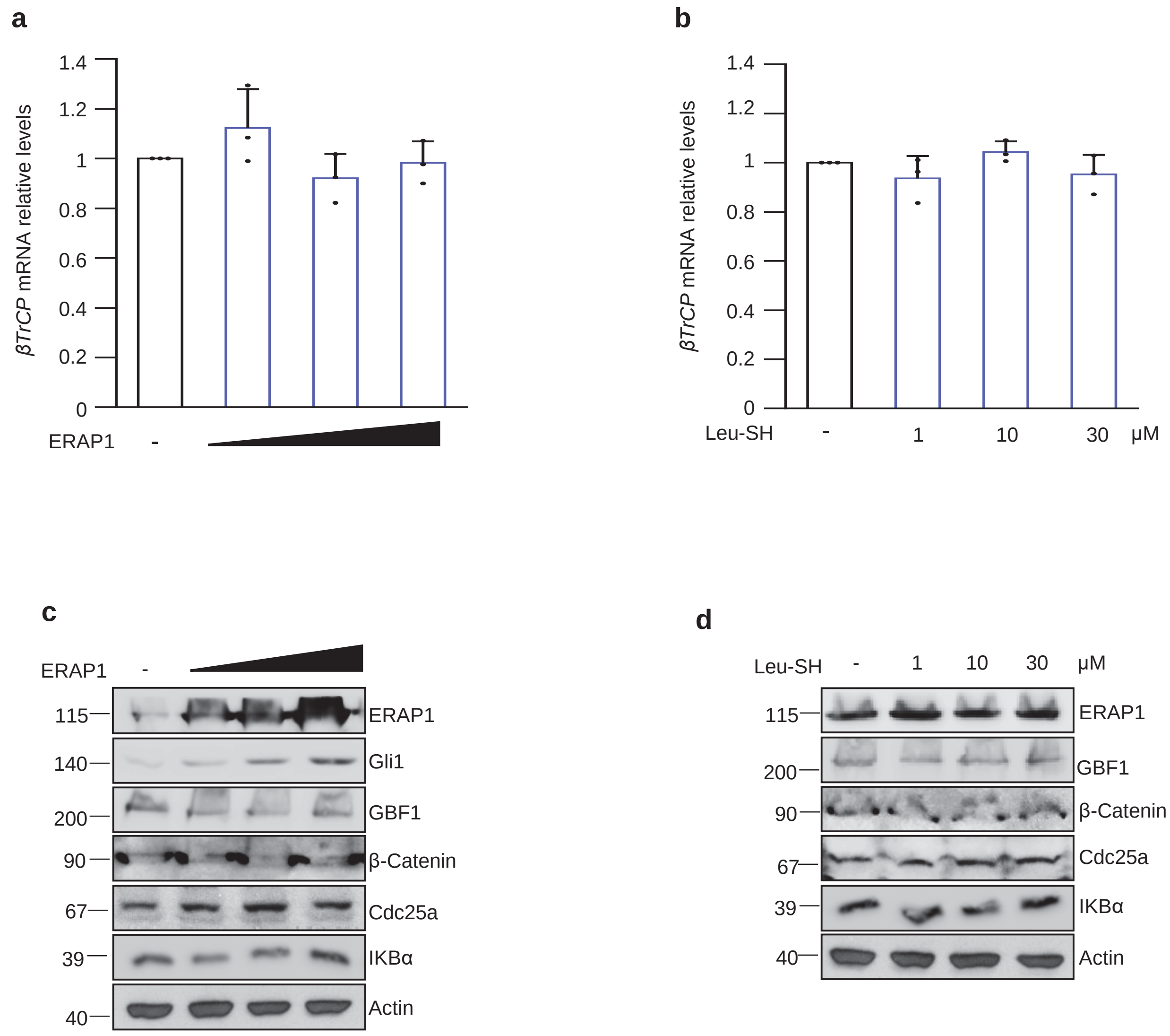

Supplementary Figure 3. **(a,b)** qRT-PCR analysis of *βTrCP* in MEFs transfected with increasing amounts of vector encoding ERAP1 (a) or treated with the indicated amounts of Leu-SH (b). Data are normalized to endogenous *GAPDH* and *HPRT* controls and represent the mean of three independent experiments. Statistical significance was calculated with two-sided Student's *t*-test. **(c,d)** Representative immunoblotting analysis of the indicated βTrCP substrates in MEFs transfected with increasing amount of vector encoding ERAP1 (c) or treated for 24 hours with Leu-SH at the indicated concentrations (d). ERAP1 expression was also evaluated and actin was used as loading control.

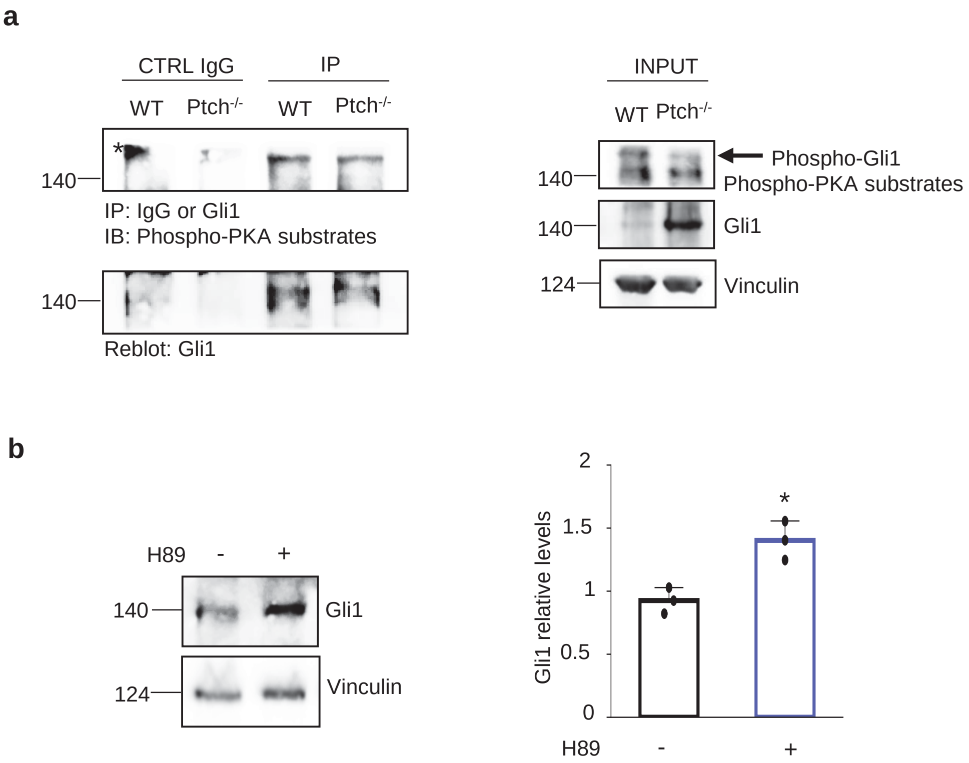

Supplementary Figure 4. (a) Cell lysates of WT or Ptch$^{-/-}$ MEFs were immunoprecipitated with an anti-Gli1 antibody and immunoblotted with anti-phospho-PKA substrates antibody. Blot was reprobed with an anti-Gli1 antibody. *Unspecific band. **(b)** Ptch$^{-/-}$ MEFs were treated for 6 hours with 30 µM H89 or vehicle. Gli1 expression was evaluated and vinculin was used as loading control. Densitometry analysis of vinculin-normalized of Gli1 values of three independent experiments was shown (rigth panel). Mean ±S.D. *P < 0.05 determined by two-tailed Student's *t*-test.

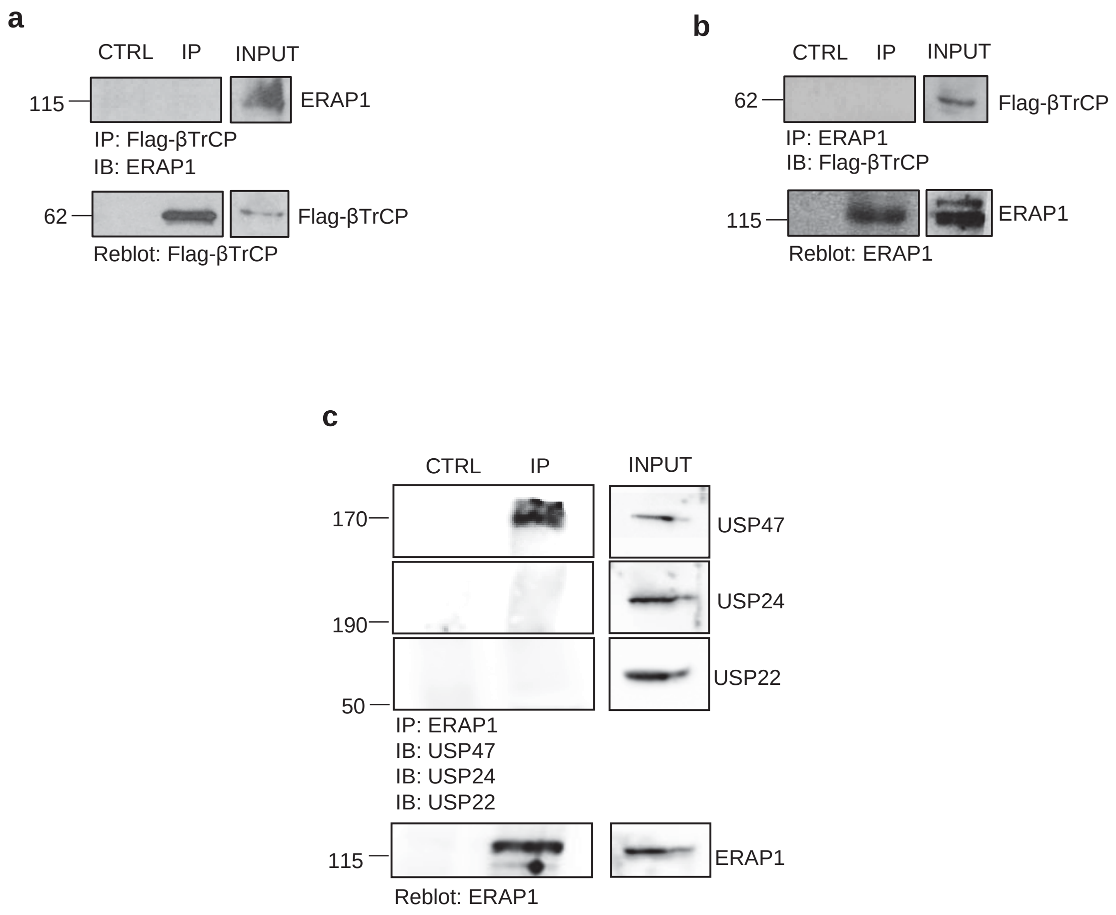

Supplementary Figure 5. (a,b) MEFs were transfected with ERAP1 and Flag-βTrCP and after 24h treated with MG132 for 4h. Cell lysates were immunoprecipitated with an anti-Flag (a) or ERAP1 antibody (b) and immunoblotted with anti-ERAP1 or anti-Flag antibody. Blots were reprobed with an anti-Flag antibody (a) or anti-ERAP1 antibody (b). **(c)** MEFs were transfected with ERAP1 and cell lysate was immunoprecipitated with an ERAP1-antibody and immunoblotted for the indicated proteins. Blots were reprobed with an anti-ERAP1 antibody.

a

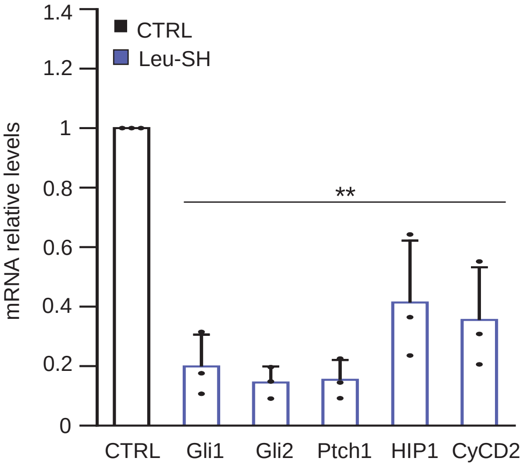

b

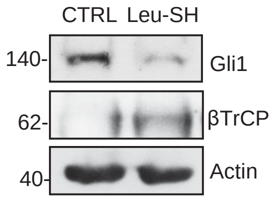

Supplementary Figure 6. **(a)** qRT-PCR analysis of Hh target genes in MB samples (pools of 3 samples for each experimental group) excised from Gfap-Cre/Ptc$^{fl/fl}$ mice treated for two days with Leu-SH or vehicle as indicated in Methods. **(b)** Immunoblotting Gfap-Cre/Ptc$^{fl/fl}$ mice analysis of Gli1 and βTrCP in MB samples from (a). Actin was used as loading control. Mean ±S.D. **P < 0.01 calculated using two-tailed Student's *t*-test.

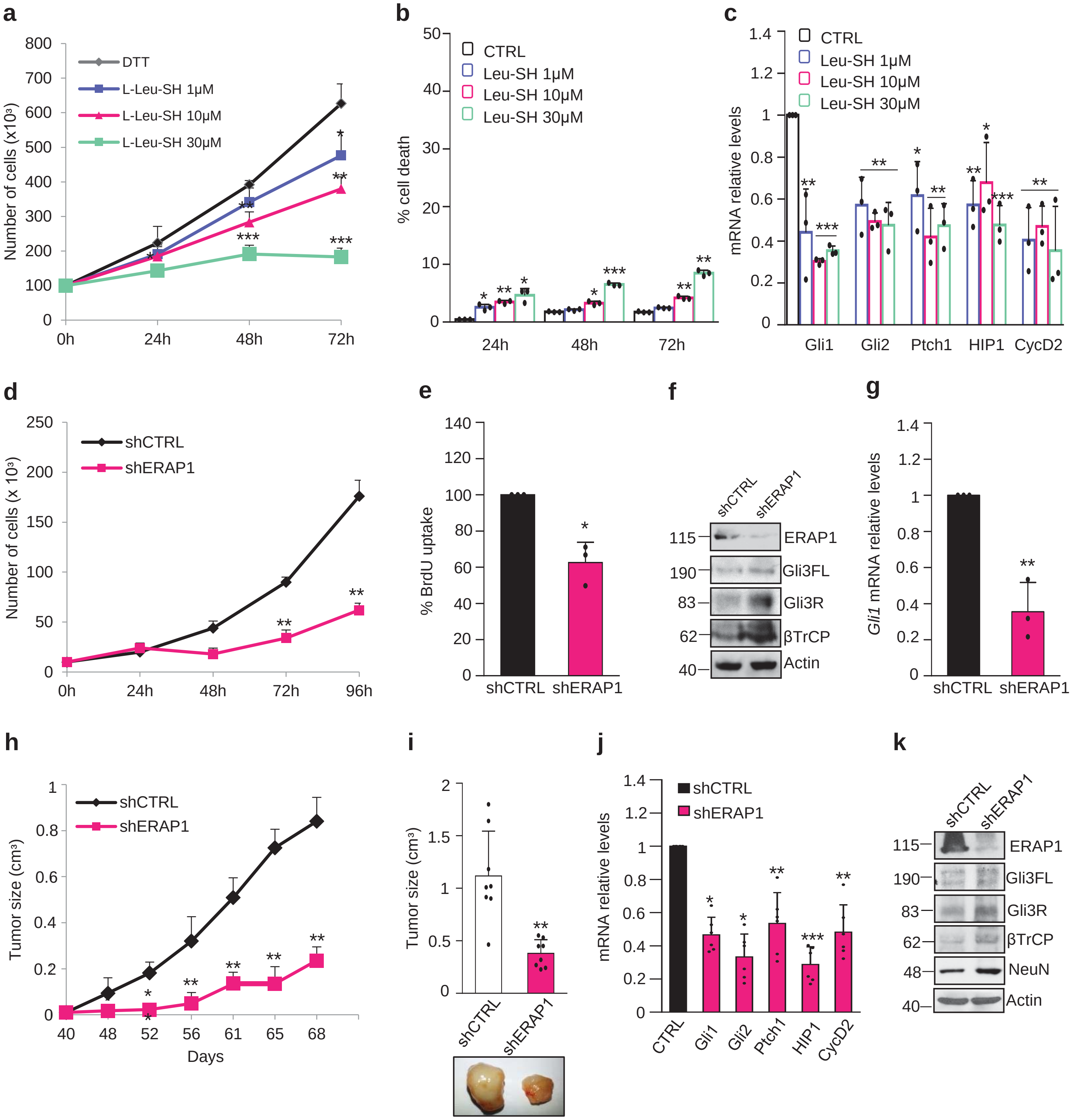

Supplementary Figure 7. ERAP1 affects Daoy cell growth both *in vitro* and *in vivo* .**(a,b)** Daoy cells were treated with Leu-SH at the indicated concentrations. Cells were counted with trypan blue at different time point to evaluate the viable cells growth rate (a) and the cell death percentage (b). **(c)** mRNA expression levels of Hh target genes of Daoy cells treated with Leu-SH at the indicated concentrations for 48h. **(d-g)** Daoy cells transduced with lentiviral particles encoding shCTRL or shERAP1. After 72h of infection cells were plated and counted with trypan blue at the indicated time points (d). Percentage of BrdU uptake (e), expression levels of the protein involved in the Hh pathway regulation (f) and *Gli1* mRNA expression level expresssed as fold change respect to the control value (g). **(h-k)** NSG mice (n=8 for group) were grafted with Daoy genetically silenced for ERAP1 expression by lentiviral infection. Tumor growth was monitored over time (h). Representative flank xenograft average volumes (lower panel) and quantification of explanted tumors (upper panel) were shown in (i). mRNA of Hh target genes (j) and protein of Gli3 and βTrCP (k) expression levels from tumor masses were assayed. Mean ±S.D. *P < 0.05; **P < 0.01; ***P < 0,001 determined by two-sided Student's *t*-test.

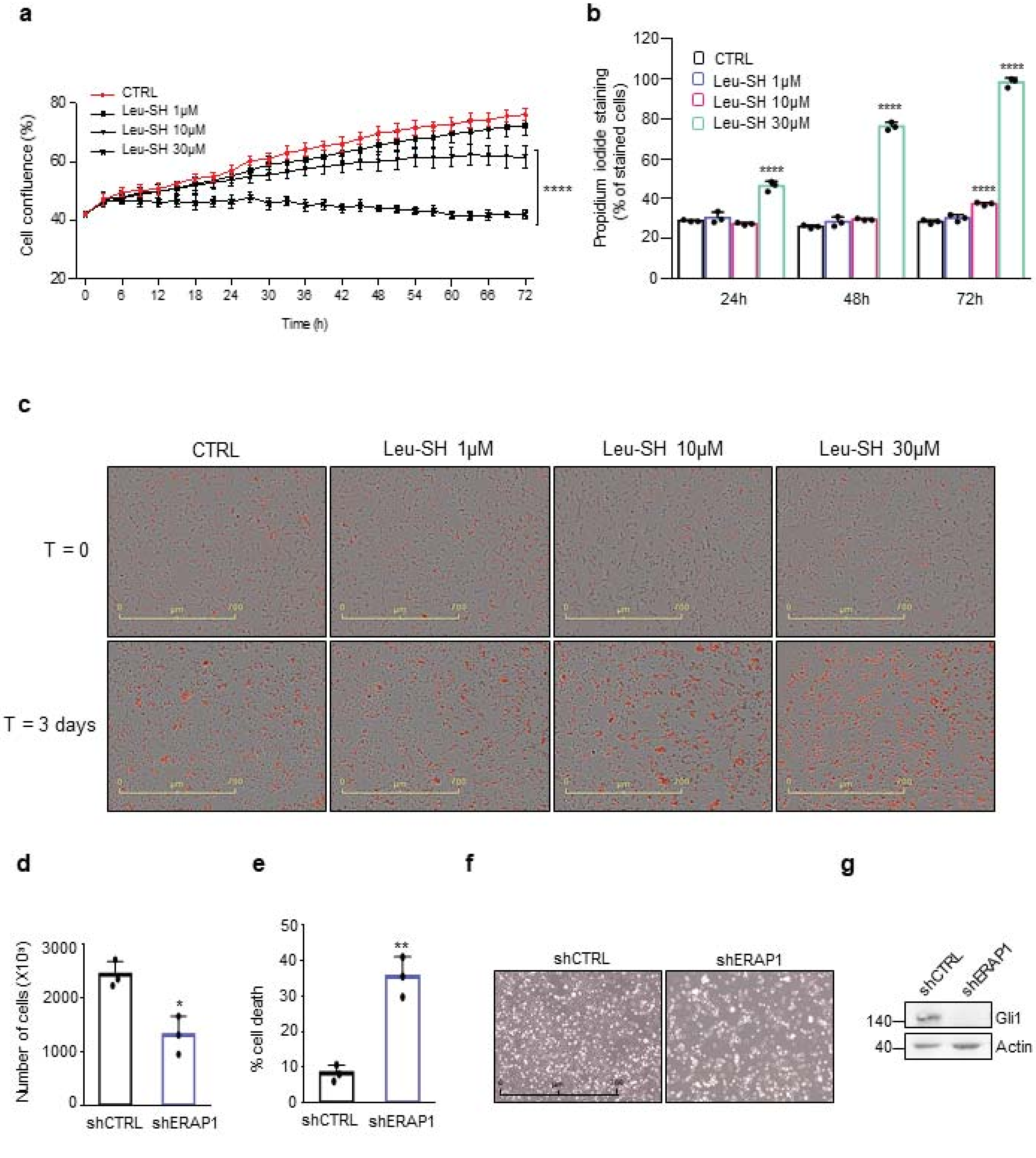

Supplementary Figure 8. **(a-c)** SHH-MB PDX tumor cells were treated at the indicated concentrations of Leu-SH for 72h. Cell confluence was monitored by IncuCyte live-imaging system (a) and cell death was assessed using propidium uptake over time (b). Representative images of the propidium iodide staining cells (in red) before and after 3 days of Leu-SH treatment are shown in (c). **(d-g)** SHH-MB PDX tumor cells were infected with lentiviral particles encoding shCTRL or shERAP1 for 72 hours. After infection, cells were counted with trypan blue to evaluate the viable cells (d) and the cell death percentage (e). Representative image of the infected cell confluence (f). Gli1 expression levels were assayed (g). Mean ±S.D. * $P < 0,05$; ** $P < 0,01$; **** $P < 0,0001$ calculated using two-tailed Student's *t*-test. For IncuCyte experiments Data were analyzed with the Two-way ANOVA test and given as mean ± SD.

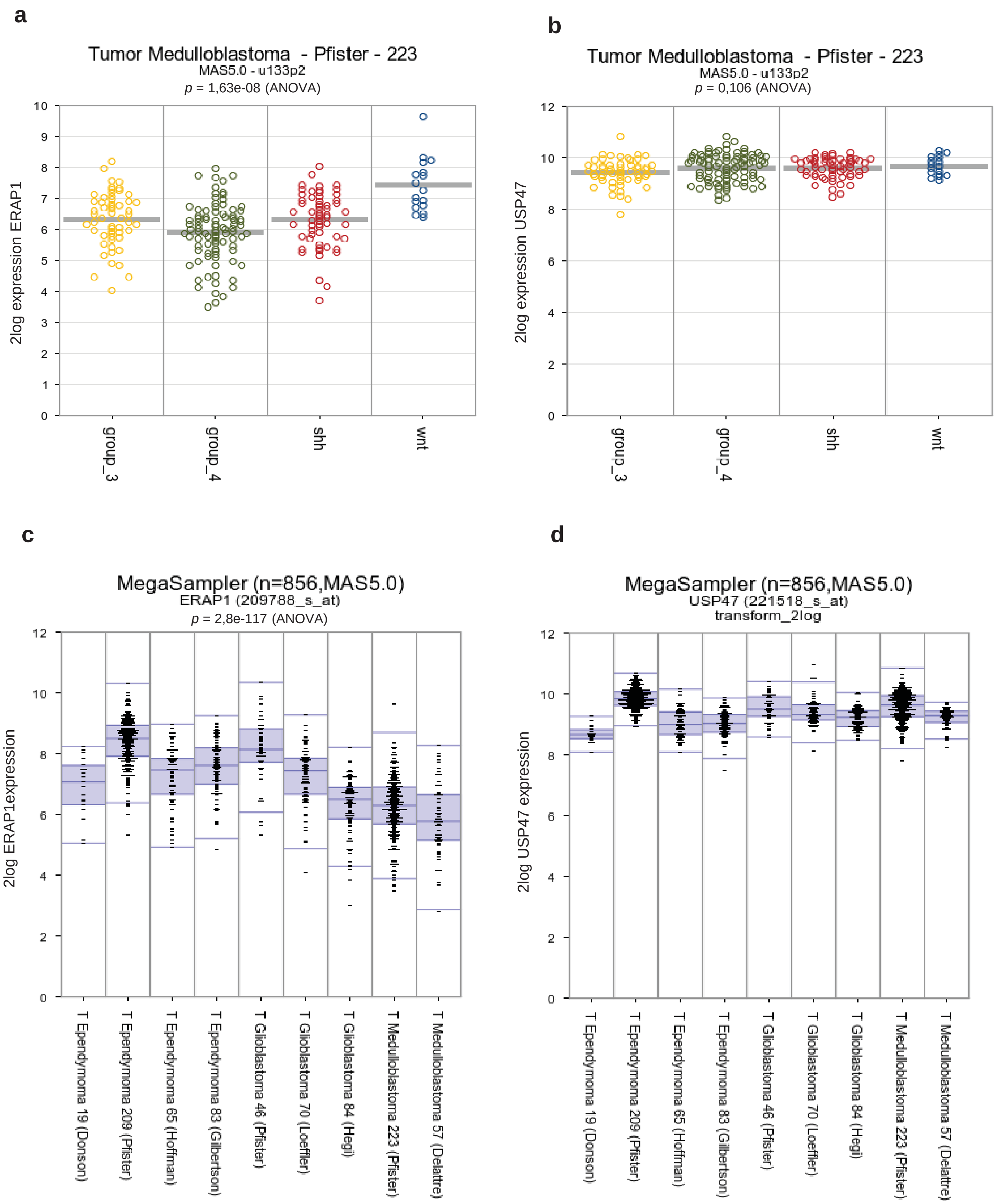

Supplementary Figure 9. (a-d) Gene expression profile of ERAP1 (a,c) or USP47 (b,d) in molecular subgroups of MB tumors (a,b) or in several data set of different brain tumors (c,d). Data were retrieved from R2 Genomics Analysis and Visualization Platform (http://r2.amc.nl).

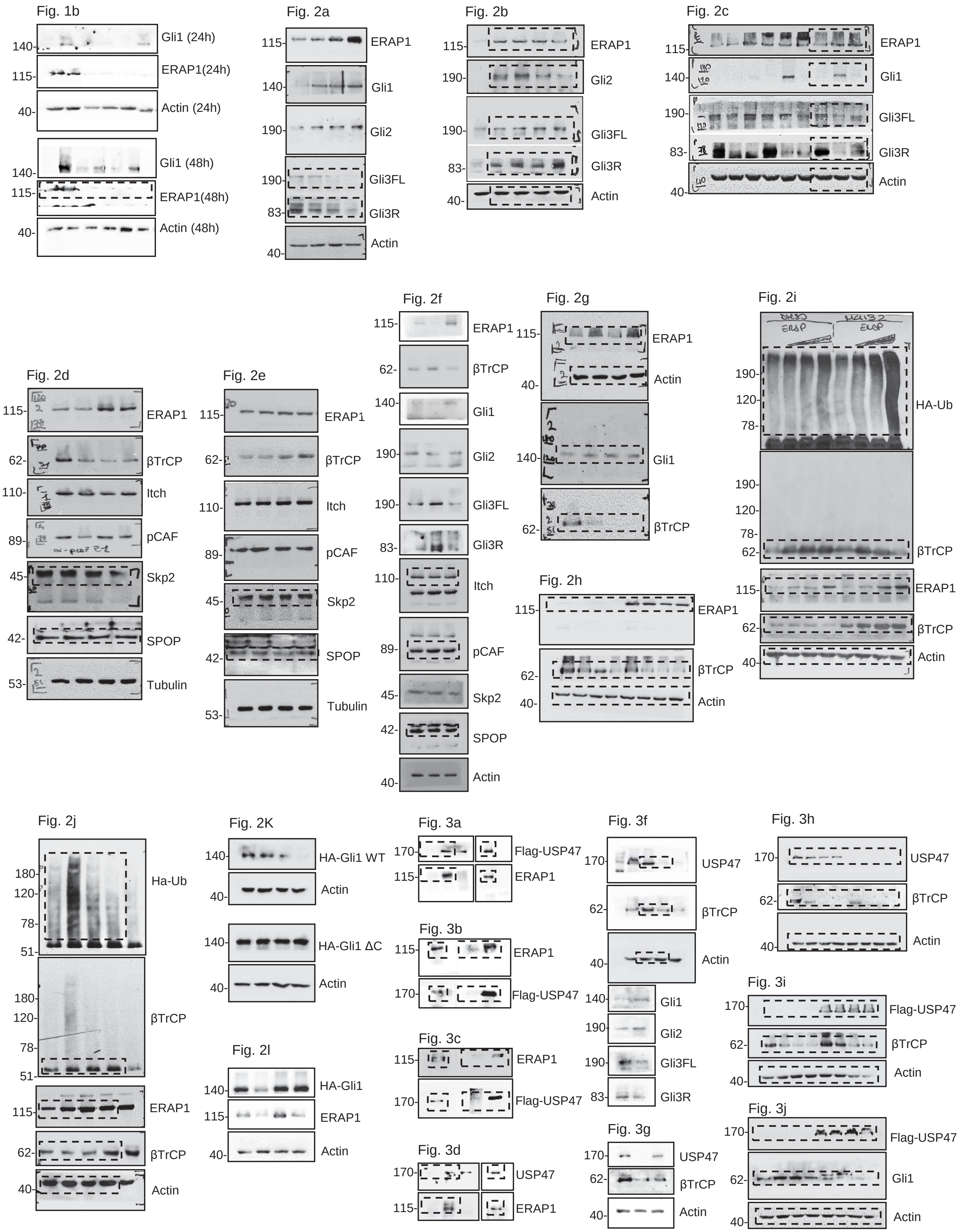

Supplementary Figure 10

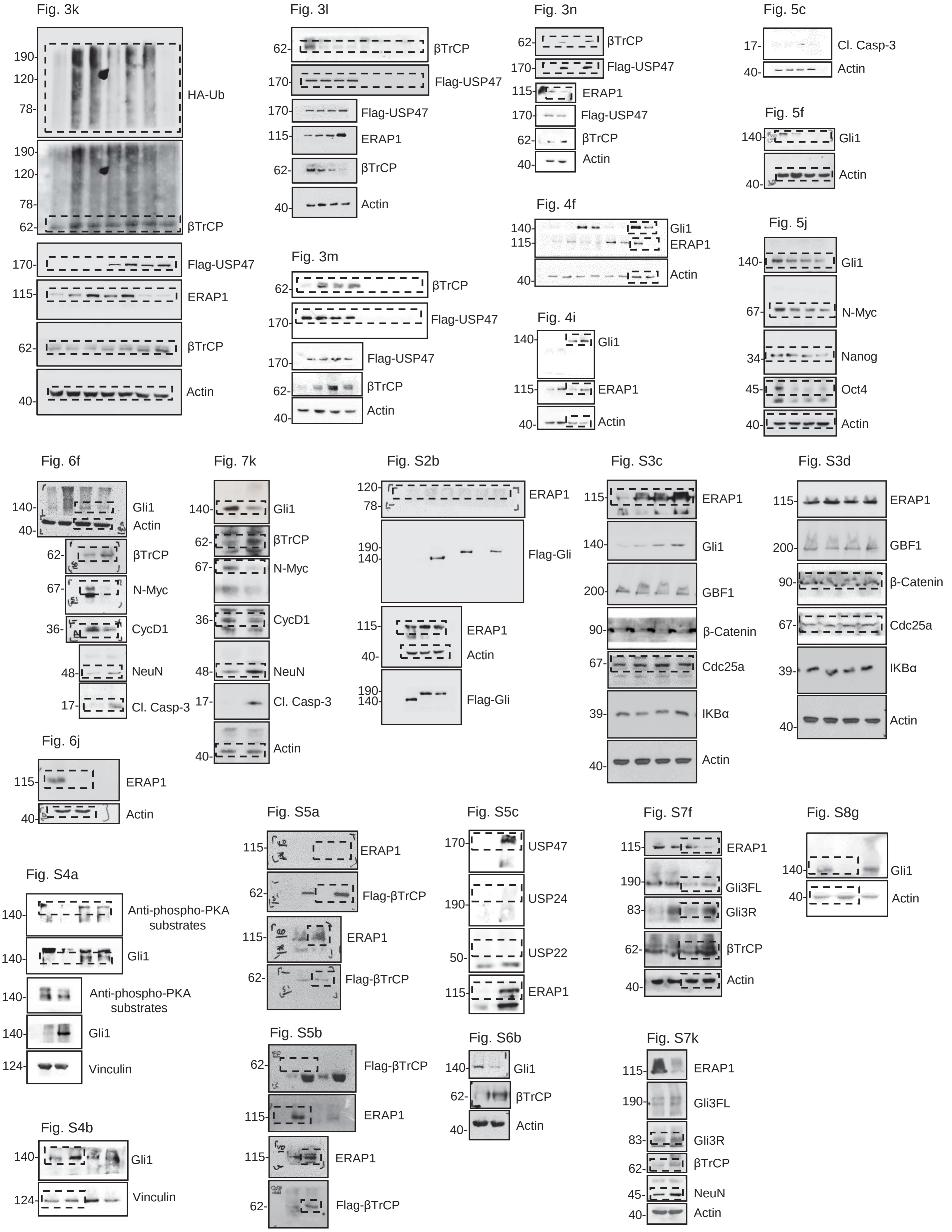

Supplementary Figure 11
